# HMGB1, anti-HMGB1 antibodies, and ratio of HMGB1/anti-HMGB1 antibodies as diagnosis indicator in fever of unknown origin

**DOI:** 10.1038/s41598-021-84477-2

**Published:** 2021-03-03

**Authors:** Mingkun Chen, Li Zhu, Miao Xue, Rongrong Zhu, Liling Jing, Huaizhou Wang, Yanghua Qin

**Affiliations:** 1 Department of Laboratory Medicine, Changhai Hospital, SMMU, Shanghai, China; 2 Department of Laboratory Medicine, Wuxi People’s Hospital, Wuxi, China

**Keywords:** Diagnostic markers, Immunological disorders

## Abstract

To evaluate the feasibility of serum HMGB1, anti-HMGB1 antibodies, and HMGB1/anti-HMGB1 ratio as a diagnosis indicator of initial clinical classification in patients with fever of unknown origin (FUO). Ninety-four patients with classical FUO and ninety healthy controls were enrolled in this study. The subjects’ clinical data and serum were collected. The serum concentration of HMGB1 was detected by a commercial HMGB1 ELISA kit, while the serum concentration of anti-HMGB1 antibodies were detected by an in-house built anti-HMGB1 antibodies ELISA kit and further confirmed by immunoblotting. According to the hospital diagnosis on discharge, ninety-four FUO patients were divided into four groups, Infectious disease subgroup, autoimmune disease subgroup, malignant tumor subgroup, and undetermined subgroup. The concentrations of HMGB1 in the infectious disease subgroup and autoimmune disease subgroup were higher than those in the malignant tumor subgroup, undetermined subgroup, and healthy control group. The concentration of anti-HMGB1 antibodies in autoimmune disease subtype group was higher than those in other subgroups as well as healthy control group. According to the distribution of HMGB1 and anti-HMGB1 in scatter plots of the patients with FUO, we found that the ratio of serum HMGB1/anti-HMGB1 is an ideal clinical indicator for differential diagnosis of different subtypes of FUO. The best cut-off was 0.75, and the sensitivity, specificity, and AUC were 66.67%, 87.32%, and 0.8, respectively. Correlation analysis showed that serum concentration of HMGB1 was moderately correlated with CRP in infectious diseases subgroup, and the serum concentration of anti-HMGB1 antibodies was strongly correlated with erythrocyte sedimentation rate in autoimmune disease subgroup. Our study had showed that serum HMGB1/anti-HMGB1 antibodies ratio can help clinicians identify FUO subtypes, thereby avoiding many unnecessary examinations and tests, and improving the effectiveness of clinical diagnosis and treatment of FUO.

## Introduction

Fever of unknown origin (FUO) was first described by Petersdorf and Beeson in 1961 as a temperature above 38.3 °C on several occasions over a period of more than 3 weeks, for which no diagnosis has been reached despite 1 week of inpatient investigation^1^. Although with more and more new medical examination and detection methods has been applied in clinical practice, FUO remains one of the most difficult diagnostic challenges in clinic. Due to more than 200 different malignant/neoplastic, infectious, rheumatic/inflammatory, and miscellaneous disorders that can cause FUO, clinicians often request many non-clue-based imaging and laboratory tests in the early stages of diagnosis and treatment, causing inconvenience and increasing the financial burden of the patient^2^.

High mobility group box 1 (HMGB1) protein was first identified as an abundant nuclear protein. After being found as a late mediator in a mice endotoxemia model^3^, the extracellular functions of HMGB1 as a mediator in sepsis have become a research highlight^4^. Now researchers believe that HMGB1 plays an important physiological role both in and out of the cell. As an alarm, extracellular HMGB1 is a damage-associated molecular pattern (DAMP) which is involved in the pathogenesis of various autoimmune diseases as well as inflammatory diseases^5,6^. In recent years, many studies, including our previous studies, have indicated that HMGB1 is closely related to the pathogenesis of chronic inflammatory diseases (e.g., tuberculous meningitis) and autoimmune diseases [especially systemic lupus erythematosus (SLE), rheumatoid arthritis, etc.]^7–9^. As an immunomodulatory protein, HMGB1 maintains the body’s immune homeostasis by altering its redox state: oxidized HMGB1 inhibits inflammatory reactions and promotes apoptosis, while reduced HMGB1 promotes inflammatory reactions and even autoimmune reactions. The elevated level of HMGB1 in serum and/or other body fluids (e.g., joint synovial fluid, urine, and cerebrospinal fluid) is often shown in patients with chronic inflammatory disease. The extracellular HMGB1 plays a pathogenic role in promoting secretion of inflammatory and chemokines, and excessive inflammation may further lead to autoimmune response and autoantibody production. No matter whether HMGB1 is oxidized or reductive, or DNA-bound or free, it is a highly sensitized autoantigen that can stimulate the body to produce corresponding autoantibodies.

Immune balance is a guarantee of human body's health. If the immunity is too weak, it can easily cause infection and tumor; if it is too strong, it can lead to autoimmune reaction and autoimmune disease. Since HMGB1 plays a key role in maintaining immune balance in the body, the imbalance of immune in patients with FUO may result in elevated levels of HMGB1 protein or anti-HMGB1 antibodies in the blood. In this study, we evaluated the potential clinical value of serum concentrations of HMGB1 and anti-HMGB1 antibodies as the first line laboratory tests in patients with FUO.

## Methods

### Data collection

Ninety-four patients diagnosed as classical FUO and ninety healthy controls were non-selectively enrolled from February 2017 to July 2018 in Changhai Hospital. The diagnostic criteria for FUO in this study were as follows: (1) body temperature > 38.3 °C (> 101 °F) in multiple checks, (2) fever time > 3 weeks, (3) hospitalization for more than 3 days, (4) the diagnosis remained unclear, after careful investigation on medical history, physical examination, and routine laboratory and imaging examinations. Exclusion criteria were: (1) iatrogenic FUO, (2) HIV-infected patients, (3) the patients with a definite diagnosis of malignant tumor, and (4) patients undergoing hormone or immunosuppressive therapy. The clinical data of the subjects, including age, sex, etc., and their serums were collected. All the samples were stored at in the − 80 °C refrigerator for the following tests. This study was exempted from requiring informed consent from the subjects as it only used the remaining serum from the clinical laboratory tests. The study was reviewed and approved by the Medical Ethics Committee of Changhai Hospital, and all the procedures followed the guidelines of the Helsinki Declaration during the study.

### Detection of serum HMGB1 and anti-HMGB1 antibodies concentrations by enzyme-linked immunosorbent assay (ELISA)

The serum concentration of HMGB1 was detected by a commercial HMGB1 ELISA kit (Arigo, Taiwan, China), while the serum concentration of anti-HMGB1 antibodies were detected by an in-house built anti-HMGB1 antibodies ELISA kit. The serum HMGB1 concentration was measured according to the kit instruction, and the measure range is 0.3–20 ng/mL. The procedure of anti-HMGB1 antibody test was as follows^10^: Briefly, Maxisorp polystyrene 96-wells plates were coated overnight at 4 °C with 50 μL per well of PBS solution containing 1 µg/mL rHMGB1 (R&D Systems, Minneapolis, USA). After one wash, plates were blocked with Blocker Casein (Thermo, Rockford, USA) for one hour. After the blocker solution was discarded, the 96-well plated were ready for testing. The serum samples, diluted 1:50 in sample buffer, were added in duplicate (100 μL/well) and incubated for 2 h at room temperature. After five washes, 100 μL HRP-conjugated rabbit anti-human IgG (Euroimmun, Lubeck, Germany) was added to each well and incubated for 30 min at room temperature. After washing, bound antibodies were detected using 3,3′,5,5′-tetramethylbenzidine dihydrochloride/hydrogen peroxide (TMB/H_2_O_2_). The chromogenic reaction was stopped by adding 100 μL of 0.5 M sulphuric acid, and the absorbance was measured at 450 nm on a microplate-spectrophotometer (Thermo MK3, Rockford, USA). The concentration of anti-HMGB1 antibodies levels was expressed in relative units.

### Clinical detection of C-reactive protein and erythrocyte sedimentation rate

Serum C-reactive protein (CRP) testing was performed on an IMMAGE 800 Specific Protein Analyzer (Beckman, USA) platform using rate scatter nephelometry. The linear detection range was 0.1–1, 152 mg/L. Erythrocyte sedimentation rate (ESR) was determined by infrared blocking method using a dynamic automatic blood sedimentation rate analyzer (MONITOR 100, Vital Diagnostics, Australia).

### Statistics

Age, CRPs and ESRs were expressed as median and extremes, and the differences between the two groups were compared with Mann–Whitney U-test. Serum HMGB1 and anti-HMGB1 antibodies concentrations were expressed as mean standard deviation, and the differences between different subgroups were compared with one-way variance test and pairwise comparison (ANOVA and multiple comparisons). The diagnostic efficiencies of serum HMGB1, anti-HMGB1 antibodies, and serum HMGB1/anti-HMGB1 antibodies ratio in the differential diagnosis of different subgroups of FUO were assessed using Receiver Operating Characteristic (ROC) curve analysis with Graphpad Prism 6 software. The correlation of serum HMGB1 and anti-HMGB1 antibodies with disease activity related indicators were analyzed with bivariate correlation analysis. The correlation coefficient r between 0.8 and 1 was considered as very strongly correlated, r between 0.6 and 0.8 was considered as strongly correlated, and r between 0.4 and 0.6 was considered as moderate correlation, r between 0.2 and 0.4 was considered as weakly correlated, and r between 0 and 0.2 was considered as very weakly correlated or uncorrelated.* P* value less than 0.05 indicated statistical significance. The statistics was performed with SPSS Statistics 21.0 Software (IBM, New York, USA).

## Results

### Clinical baseline characteristics

The basic clinical data and laboratory results for FUO patients and healthy controls were presented in Table 1. Ninety-two of the ninety-four patients with FUO had been diagnosed with a definitive etiology when they were to be discharged from hospital and two had not been diagnosed with a definitive etiology when they were to be discharged from hospital. In this study, the top three main etiologies of FUO were infectious disease (the infectious disease subgroup, 71/94, 75.5%), autoimmune disease (the autoimmune disease subgroup, 18/94, 19.1%), and malignant tumor (3/94, 3.2%). Within the autoimmune disease subgroup, SLE accounted for 33% (6/18), followed by Sjogren syndrome (SS), adult still's disease, and systemic vasculitis (SV).

**Table 1:** Clinical characteristics and laboratory tests in FUO and health controls.

	Infectious disease (71)	Autoimmune disease (18)	Tumor (3)	Health controls (90)
Age median (range, years)	59 (23–82)	56.5 (36–76)	69 (44–76)	49 (15–78)
Male to female ratio	41:30	5:13	2:1	48:42
ESR median (range, mm/h)	13 (4–20)	27 (8–132)	8 (6–15)	–
CRP median (range, mg/L)	21.3 (3.1–218)	4.9 (0.5–12.6)	0.8 (0.5–4.6)	–
RF median (range, IU/mL)	25.4 (10.4–109.4)			–

### Serum anti-HMGB1 antibodies in patients with autoimmune diseases

In-house built Anti-HMGB1 antibodies ELISA was first used for the detection of anti-HMGB1 antibodies in three common autoimmune diseases, i.e., SLE, SS, and RA, and healthy controls (see Supplementary Information for details). The serum anti-HMGB1 antibodies were significantly elevated in 20 SLE, 12 SS, and 11 RA patients, compared with the healthy controls (Supplementary Fig. S1a, *P* < 0.01). Some of the positive results were further confirmed by immunoblotting (Supplementary Fig. S1b).

### The serum concentrations of HMGB1 and anti-HMGB1 antibodies in patients with FUO

**Figure 1:**
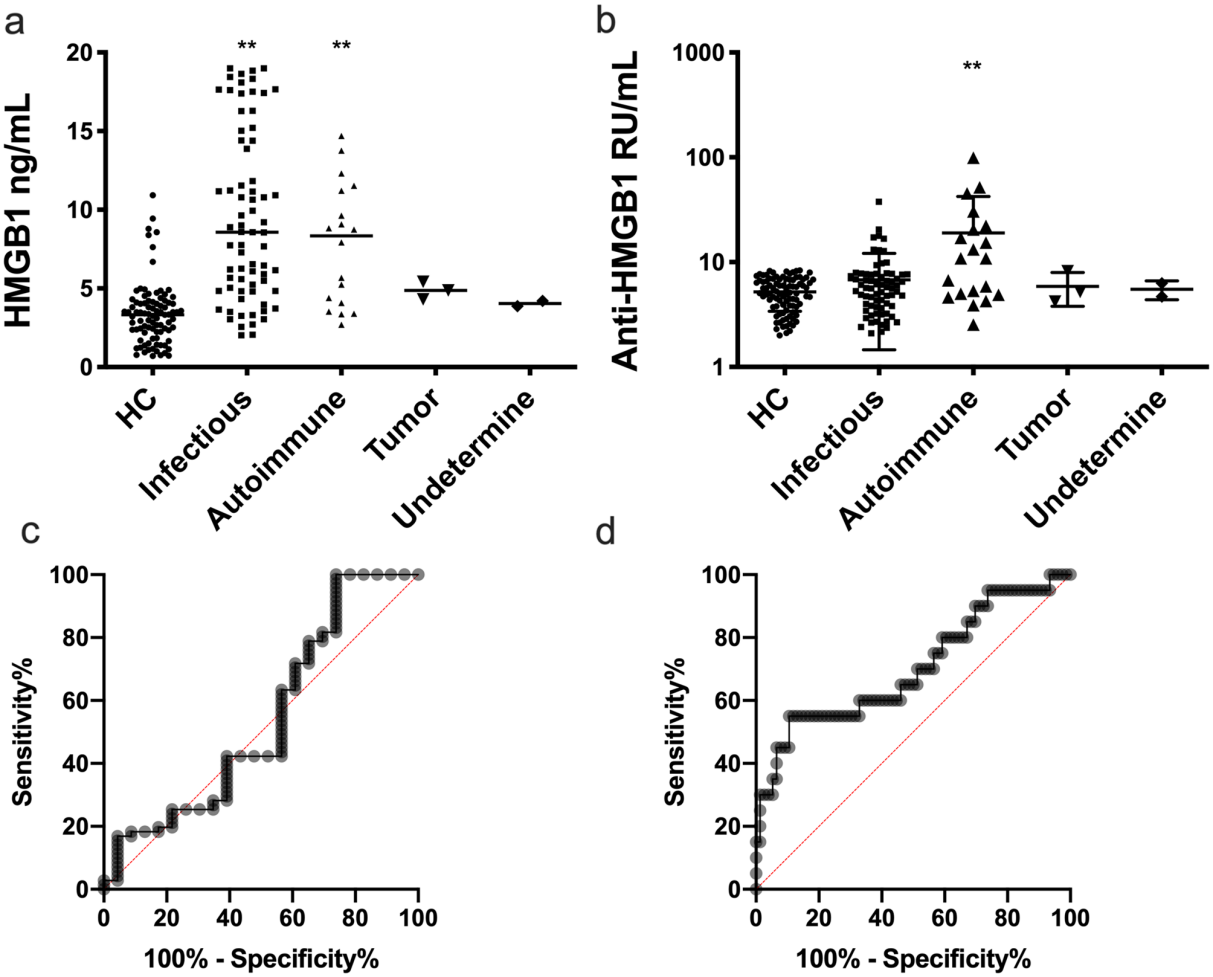
Serum HMGB1 and anti-HMGB1 antibodies in patients with FUO. (**a**) Serum HMGB1 in different subtypes of patients with FUO; (**b**) Serum anti-HMGB1 antibodies in different subtypes of patients with FUO; (**c**) ROC curve of serum HMGB1 in diagnosis of infectious disease subtype in patients with FUO; (**d**) ROC curve of serum anti-HMGB1 antibodies in diagnosis of autoimmune disease subtype in patients with FUO. (*P < 0.05; **P < 0.01 compared with HC).

The serum concentrations of HMGB1 in FUO patients and healthy controls were measured using ELISA testing kit and the results were shown in Fig. 1. The serum concentration of HMGB1 in the infectious disease subgroup and autoimmune disease subgroup were significantly higher than that of healthy control group (Fig. 1a). The serum concentration of anti-HMGB1 antibodies in the autoimmune disease subgroup was significantly elevated than those of the healthy controls and other subgroups (Fig. 1b). The diagnostic efficiencies of serum HMGB1 and anti-HMGB1 antibodies in differentiating the infectious disease subgroup from the autoimmune disease subgroup was assessed with ROC curve analysis. In the patients with FUO to diagnose infectious disease subtype, the most appropriate cut-off value for serum HMGB1 was 6.63 ng/mL with the sensitivity (42.3%), specificity (60.9%), and area under the curve (0.54); to diagnose autoimmune disease subtype, the most appropriate cut-off value for serum anti-HMGB1 antibodies was 9.81 RU/mL with the sensitivity (55%), specificity (88.2%), and area under the curve (0.70) (Fig. 1c,d).

### Ratio of serum HMGB1/anti-HMGB1 antibodies in patients with FUO

**Figure 2:**
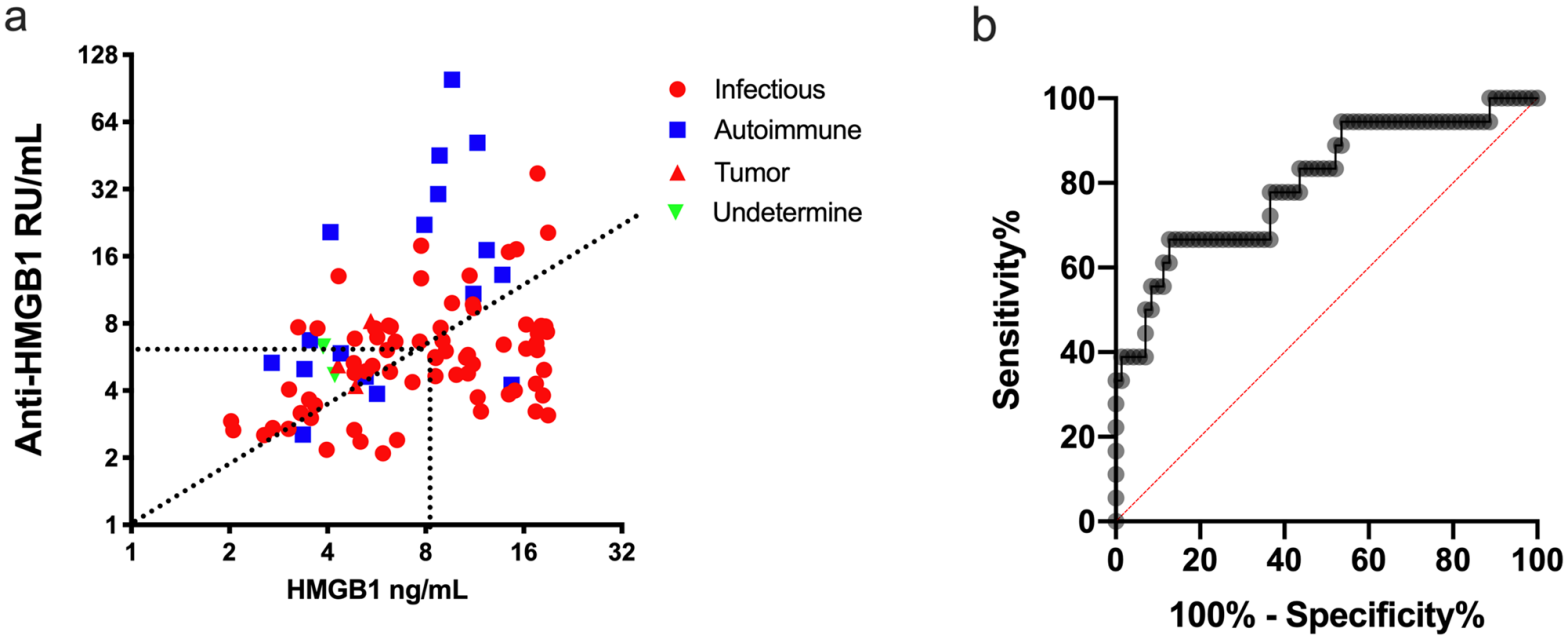
The ratio of serum HMGB1/anti-HMGB1 antibodies in patients with FUO. (**a**) The distribution of the concentrations of serum HMGB1 and anti-HMGB1 antibodies in different subtypes of patients with FUO; (**b**) ROC curve of the ratio of serum HMGB1/anti-HMGB1 antibodies in diagnosis infectious and autoimmune disease subtypes in patients with FUO.

The distribution of serum HMGB1 and anti-HMGB1 antibodies concentrations of the 92 FUO patients diagnosed with definitive etiologies (subtypes) was shown in the two-dimensional scatter plot (Fig. 2a), which showed the ratio of serum HMGB1/anti-HMGB1 antibodies had a good diagnostic value in the differential diagnosis of different subtypes of FUO, especially in the subgroups of infectious diseases and autoimmune diseases. The results of ROC curve analysis on the efficiency of the serum HMGB1/anti-HMGB1antibodies Ratio in the differential diagnosis of FUO infectious disease or autoimmune disease subtypes was shown in Fig. 2b. The best cut-off value was 0.75, and the sensitivity, specificity, and AUC were 66.67%, 87.32%, and 0.8, respectively.

### Correlation of serum HMGB1 or anti-HMGB1 antibodies concentrations with disease activity indicators

**Figure 3:**
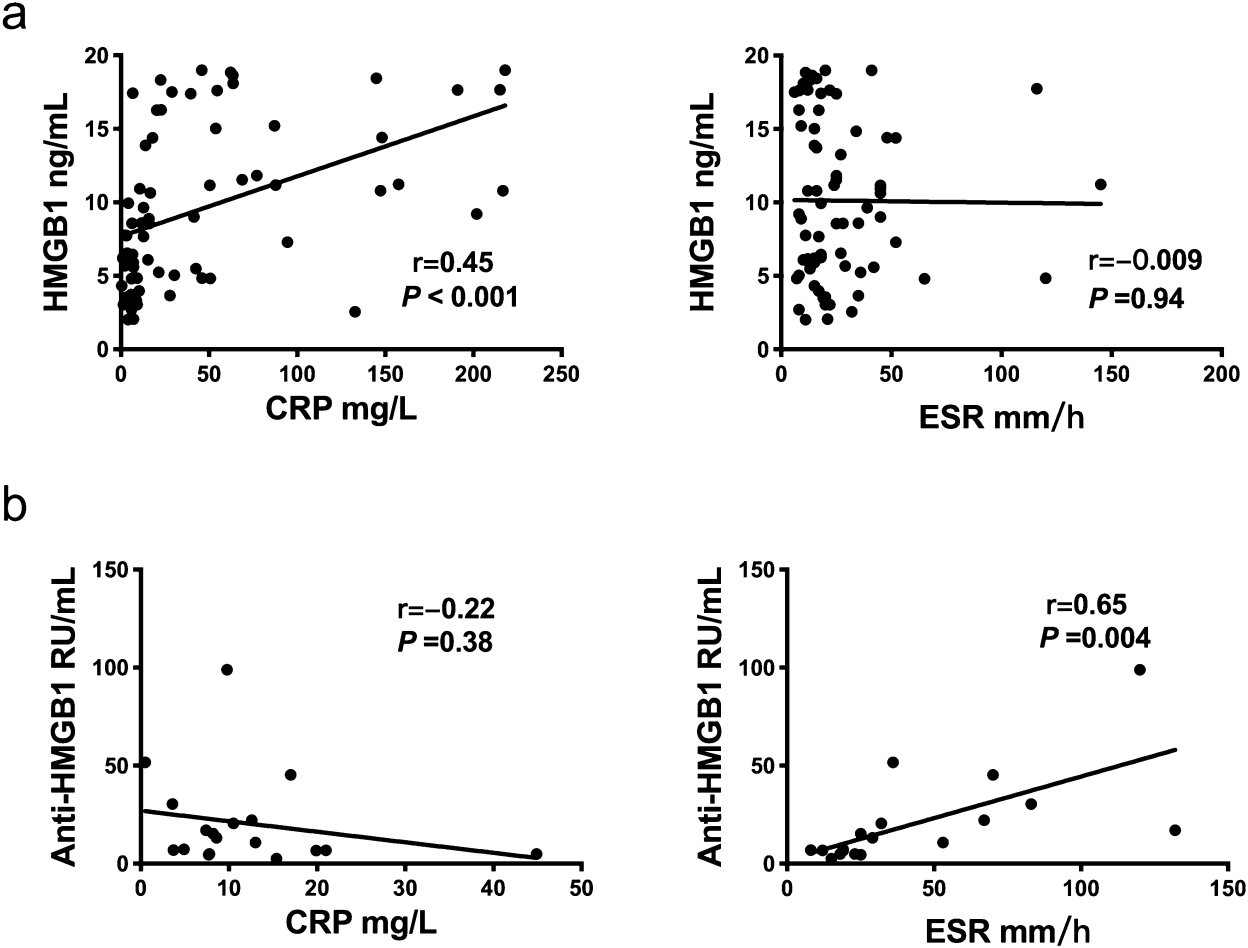
Correlation analysis of serum HMGB1 or anti-HMGB1 antibodies with disease severity markers in patients with FUO. (**a**) Serum HMGB1 is correlation with CRP and ESR in patients with FUO; (**b**) Serum anti-HMGB1 antibodies is correlation with CRP and ESR in patients with FUO.

In this study, we assessed the correlation of serum HMGB1 and anti-HMGB1 antibodies concentrations with disease activity indicators (CRP and ESR) in FUO infectious disease subgroups and autoimmune disease subgroups using the bivariate correlation analysis. The results showed that serum HMGB1 concentration moderately correlated with CRP concentration in infectious subgroup, while serum anti-HMGB1 antibodies were strongly correlated with ESR in autoimmune disease subgroup (Fig. 3).

## Discussion

In 1991, Durack et al. proposed splitting the subtype of FUO into four subtypes based on the underlying cause: classic FUO, iatrogenic FUO, immunodeficient FUO, and HIV-associated FUO^11^. Although more and more novel examinations and tests are applied in clinical practice, the diagnosis and treatments of classic FUO are still a major challenge in the modern medicine. Because of the complexity and variety of FUO-related diseases, the multitude of etiologies, and the atypical symptoms and signs, the diagnosis of the intrinsic etiologies of FUO requires extensive time-consuming physical, laboratory tests, and even diagnostic treatment. At present, with the change of disease spectrum, the development of modern diagnosis and treatment technology, and the clinical understanding of FUO, the number of FUO cases which cannot be diagnosed with definite etiology has gradually decreased. Infection is the most common and major etiology of classic FUO; abscess, endocarditis, tuberculosis, and complex urinary tract infections are the most common causes of infection us subtype in FUO^12^. With advances in imaging diagnostic techniques, many new imaging technologies, e.g., such as PET-CT and PET-MRI, have been widely used clinically, and most malignant tumor-related FUO can be further diagnosed. In patients with cancer, fever can be either a symptom of the tumor itself or indicator of complex infection. Recently, the autoimmune diseases have overtaken malignant tumors as the second most common subtype of FUO, among which SLE, RA, SS and adult still's disease are the most common diseases. Due to the lack of specific autoantibodies and other specific diagnostic criteria, the diagnosis of adult still's disease needs to exclude other possible disorders. Thus, it is time-consuming process to determine the subtype of FUO and make up the treatment plan, and there is an urgent need for a new clinical test to rapidly differentiate different subtypes of FUO.

In China, the majority of clinical FUO belong to infectious and autoimmune disease subtypes. When a clinician receives a FUO patient, the primary task is to determine whether it is an infectious subtype or an autoimmune disease subtype, and to evaluate the patient's state to decide whether to give empirical anti-infective therapy or empirical hormone anti-inflammatory therapy^13^. Therefore, how to quickly determine etiology, i.e., whether it is an infectious disease or autoimmune disease, is the key to diagnosis and treatment of FUO. HMGB1 can stimulate immune cells to migrate to the injured sites, promote the human body to recognize bacteria and activate innate immune cells, produce pro-inflammatory cytokines and aggravate inflammatory response, and inhibit the apoptosis of neutrophil. Many researches have shown that serum HMGB1 is a potential biomarker of infectious disease, such as pneumonia^14,15^, hand-foot-mouth disease^16^, hemorrhagic fever with renal syndrome^17^, and scrub typhus^18^. Sobajima et al.^19^ first described Anti-HMGB1 antibodies as a perinuclear anti-neutrophil cytoplasmic antibody (pANCA) in ulcerative colitis. HMGB1 is a component of nucleosome complex, and nucleosome is a large group source of auto-antigens. At present, anti-HMGB1 antibodies are generally considered to be related to autoimmune dysfunction, but the role of HMGB1/anti-HMGB1 antibodies in the occurrence and development of autoimmune diseases has not been fully elucidated. Studies have shown that anti-HMGB1 antibodies can reduce the pathogenic effect of HMGB1 by blocking the inflammatory effects of HMGB1 in animal models of arthritis^20^ and Sjӧgren's syndrome^21^. The use of the neutralizing antibody anti-HMGB1 in the treatment of lupus-prone BXSB mice can reduce the concentration of proteinuria, glomerulonephritis, circulating anti-dsDNA, immune complex deposition, and plasma cytokines^22^. HMGB1 can also be used as an autoantigen to directly stimulate the body to produce anti-HMGB1 autoantibodies. Some studies reported that anti-HMGB1 could be detected in peripheral blood of patients with SLE, and the concentration of anti-HMGB1 antibodies was positively correlated with the SLE disease activity score (SLEDAI) and degree of renal injury^10,23–26^. However, the biological/clinical significance of anti-HMGB1 antibodies has not been full elucidated.

In this study, we sought to differentiate different FUO subtypes by serum HMGB1 and anti-HMGB1 antibodies. Our results showed that the serum concentrations of HMGB1 in peripheral blood of FUO patients with infectious and autoimmune subtypes were significantly increased, and the concentrations of HMGB1 in serum of patients with infectious subtype were positively correlated with CRP. The serum concentrations of anti-HMGB1 antibodies in the patients of autoimmune disease subtype were significantly higher than those of the other (infectious, malignant tumor-related, and undetermined) subtypes, and the concentrations of anti-HMGB1 antibodies in serum of patients with autoimmune disease subtype were positively correlated with ESR. With ROC analysis, we showed that the concentrations of serum HMGB1 have certain differential diagnostic value in the differential diagnosis of infectious subtype of FUO and the concentrations of serum anti-HMGB1 antibodies have certain differential diagnostic value in the differential diagnosis of autoimmune disease subtype of FUO. Based on the distribution of different subtypes in the two-dimension scatter plots, we found that the ratio of serum HMGB1/anti-HMGB1 antibodies appeared to be a good diagnostic marker for differentiate the subtypes of FUO. By ROC curve analysis, we confirmed that the ratio of serum HMGB1/anti-HMGB1 antibodies is a good initial laboratory test for patients with FUO.

## Conclusion

In summary, as an immunomodulatory protein, HMGB1 plays a key role in both inflammatory and autoimmune responses, and our study indicates that the ratio of serum HMGB1/anti-HMGB1 antibodies is a suitable laboratory test of choice for clinically differentiating the subtypes of FUO.

## Supplementary Information

Supplementary Figure S1.

Supplementary Information.
