# Supplementary figures and images for: HMGB1, anti-HMGB1 antibodies, and ratio of HMGB1/anti-HMGB1 antibodies as diagnosis indicator in fever of unknown origin
Source: Sci Rep. 2021 Mar 3;11:5059. doi: 10.1038/s41598-021-84477-2 (PMC7930274; doi:10.1038/s41598-021-84477-2)

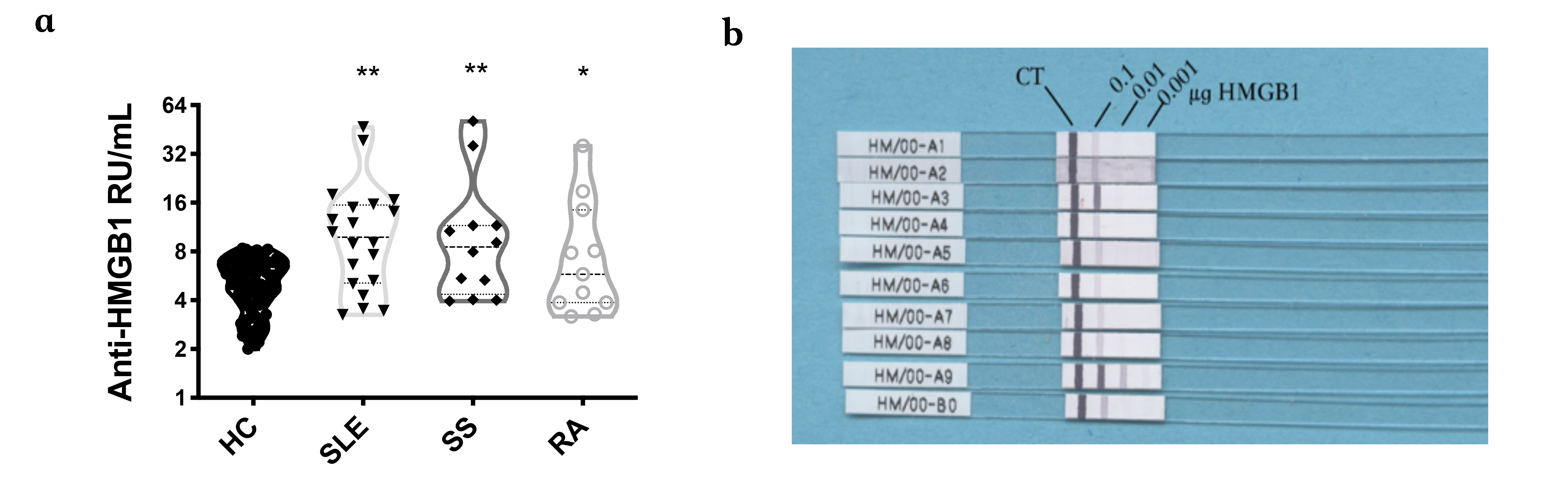

Supplement: Supplementary file 1 — Supplementary Figure S1. [file 41598_2021_84477_MOESM1_ESM.tif]
