# Supplementary material for: HMGB1, anti-HMGB1 antibodies, and ratio of HMGB1/anti-HMGB1 antibodies as diagnosis indicator in fever of unknown origin
Source: Sci Rep. 2021 Mar 3;11:5059. doi: 10.1038/s41598-021-84477-2 (PMC7930274; doi:10.1038/s41598-021-84477-2)
Supplement: Supplementary file 2 — Supplementary Information. [file 41598_2021_84477_MOESM2_ESM.docx]

HMGB1, anti-HMGB1 antibodies, and ratio of HMGB1/anti-HMGB1 antibodies as diagnosis indicator in fever of unknown origin

Mingkun Chen^1, #^, Li Zhu^2, #^, Miao Xue^1, #^, Rongrong Zhu^1^, Liling Jing^1^, Huaizhou Wang^1,^*, Yanghua Qin^1,^*

**1** Department of Laboratory Medicine, Changhai Hospital, SMMU, Shanghai, China

2 Department of Laboratory Medicine, Wuxi People’s Hospital, Wuxi, China

Correspondence should be addressed to Yanghua Qin: qinyanghua@smmu.edu.cn or Huaizhou Wang: whz_sh@163.com

ORCID of Yanghua Qin: 0000-0001-9516-7441

Supplementary methods:

1. Patients: 11 RA, 12 SS, and 20 SLE out patients from February 2017 to July 2018 in the Changhai Hospital were enrolled in this research. The diagnosis of RA, SS, and SLE meets with the criteria from the Chinese Rheumatology Association.

2. Immunoblotting

The serum anti-HMGB1 antibody positive samples were further confirmed by an in-house built immunoblotting: Nitrocellulose membrane was coated with three bands of different amount of rHMGB1 protein (0.001, 0.01, and 0.1μg). The strips were incubated with 15μl serum for 2 hours at room temperature. After 3 washes, ALP-conjugated goat anti-human IgG (Euroimmun, lubeck, Germany) was added to each lane and incubated for 30 minutes at room temperature. After washing, bound antibodies were detected using nitro blue tetrazolium/ 5-bromo-4-chloro-3-indolyl phosphate (NBT/BCIP). The bands in the strip were detected with a scanner and analyzed with EUROLineScan.
